# Gender-specific prevalence and associated factors of major depressive disorder and generalized anxiety disorder in a Chinese rural population: the Henan rural cohort study

**DOI:** 10.1186/s12889-019-8086-1

**Published:** 2019-12-27

**Authors:** Zhicheng Luo, Yuqian Li, Yitan Hou, Xiaotian Liu, Jingjing Jiang, Yan Wang, Xue Liu, Dou Qiao, Xiaokang Dong, Ruiying Li, Fang Wang, Chongjian Wang

**Affiliations:** 1 Department of Epidemiology and Biostatistics, College of Public Health, Zhengzhou University, 100 Kexue Avenue, Zhengzhou, 450001 People’s Republic of China; 2 Department of Clinical Pharmacology, School of Pharmaceutical Science, Zhengzhou University, Zhengzhou, Henan People’s Republic of China; 3 Department of Global Health, School of Health Sciences, Wuhan University, Wuhan, Hubei China; 4 Department of Epidemiology, School of Public Health, Shanxi Medical University, Taiyuan, Shanxi People’s Republic of China

**Keywords:** Major depressive disorder, Generalized anxiety disorder, Prevalence, Associated factors, Rural population

## Abstract

**Background:**

This study aims to investigate the prevalence and associated factors of major depressive disorder (MDD) and generalized anxiety disorder (GAD) by gender in Chinese rural adults.

**Methods:**

A total of 29,993 participants aged from 18 to 79 years from the Henan Rural Cohort Study were included in this study. The Patient Health Questionnaire-2 (PHQ-2) and Generalized Anxiety Disorder-2 (GAD-2) were used to assess MDD and GAD through a face-to-face interview. Multivariate logistic regression model was conducted to analyze the associated factors for MDD and GAD.

**Results:**

The age-standardized prevalence of MDD and GAD (and 95%*CI*) in the total sample were 5.41% (5.17–5.66%) and 4.94% (4.71–5.18%), respectively. Besides, the crude prevalence in women were significantly higher than men for both MDD (6.81% vs. 4.77%) and GAD (6.63% vs. 3.93%) (both *P* < 0.001). Tetrachoric correlation test showed high comorbidity between MDD and GAD (*r* = 0.88, *P* = 0.01). Further analysis revealed that age, sex, marital status, educational level, per capita monthly income, drinking, physical activity, and body mass index were associated with MDD and GAD in the overall sample. Gender difference was found among age groups for MDD (*P*_*interaction*_ < 0.001).

**Conclusions:**

These findings showed that Chinese rural adults were at low risk for prevalence of MDD and GAD. Women had higher prevalence and risks for MDD and GAD compared with men, indicating that women deserved more attention. Gender-specific interventions on the modifiable associated factors are urgently needed to improve the mental conditions for Chinese rural population.

**Clinical trial registration:**

The Henan Rural Cohort Study has been registered in the Chinese Clinical Trial Register (Registration number: ChiCTR-OOC-15006699). Date of registration: 2015-07-06.

## Background

According to the World Health Organization, by 2020, about 25% of the world’s population will suffer from mental disorders which are expected to account for 15% of the global disease burden [1]. In China, there has been a rapid increase in mental disorders since the 1990s [2], and nearly one-sixth of individuals had mental disorders by 2001–2005 [3]. Major depressive disorder (MDD) and generalized anxiety disorder (GAD), as two of the most preventable and treatable mental disorders, commonly coexist with each together [4, 5]. Previous studies showed that the prevalence of MDD and GAD were initially low but drastically increasing in the past few decades in China regardless of the different diagnostic and screening tools and criteria [6]. A large population-based survey conducted during 2001–2005, involving 113 million individuals that accounted for 12% of the Chinese adult population, showed that the prevalence of MDD and GAD were 2.07 and 1.32%, respectively [3]. These estimates were much higher than the reports in two national epidemiological surveys in the 1980s and 1990s, which showed extremely low prevalence of MDD and GAD in China [7].

Although previous studies have explored the prevalence of MDD and GAD among Chinese adults, the evidence remains limited and insufficient in the rural population [3, 6, 7]. What is more, few studies focus attention on the gender differences of MDD and GAD for Chinese. In fact, multiple studies have documented that women had higher prevalence and were approximately twice as likely to suffer from MDD and GAD as men [8–10]. Besides, men and women had disparate cross-sectional relationships with demographic, lifestyle and other potential factors that were unique to each gender [8–10]. Therefore, it is indispensable to investigate the prevalence and associated factors of MDD and GAD in the different sexes.

The present study aimed to investigate the gender-specific prevalence of MDD and GAD in Chinese rural adults and, secondly, further explore the associated factors of MDD and GAD according to gender.

## Methods

### Study subjects

The study population was from the Henan Rural Cohort study [11], which was registered in the Chinese Clinical Trial Register before the onset of patient enrollment (Registration number: ChiCTR-OOC-15006699, http://www.chictr.org.cn/showproj.aspx?proj=11375). Briefly, the study was conducted in Yuzhou, Suiping, Tongxu, Xinxiang, and Yima counties of Henan province in China between July 2015 and September 2017. A total of 39,259 people participated in this study with a response rate of 93.7% and the age of these participants ranged from 18 to 79 years. Among the 39,259 subjects, 30,001 subjects completed the psychological questionnaire survey. Finally, 29,993 participants who completed the information of both PHQ-2 and GAD-2 questionnaires were included in the analysis. A more detailed information on the cohort has been described elsewhere [11]. The Henan Rural Cohort study was approved by the Zhengzhou University Life Science Ethics Committee (Code: [2015] MEC (S128)), and the informed consent was obtained from all participants. The study was conducted according to the Declaration of Helsinki.

### Assessment of covariates

A standardized survey questionnaire was carried out by well-trained staff through face-to-face interviews to collect detailed information on demographic characteristics, personal history of chronic diseases and medication, and lifestyle factors. Educational level was divided into primary school or below and junior high school or above. Per capita income was calculated from household income divided by the number of household members (including children). Then socioeconomic status was evaluated according to per capita monthly income (< 500, 500~, and ≥ 1000 Renminbi (RMB)). Marital status was divided into married/cohabitating and widowed/single/divorced. Smoking status was classified into current smoker (a person who smoked more than one cigarette per day in the past six months) and non-current smoker (including ever smoker and never smoker). Alcohol drinking status was categorized into current drinking (a person who consumed alcoholic drinks for twelve or more times in the past one year, whether spirits, beer, wine or other forms of alcoholic beverage), and non-current drinker (including ever drinker and never drinker). Physical activity was grouped into low, moderate and high levels according to the international physical activity questionnaire (IPAQ 2001) [12]. Information on chronic disease history was obtained by self-reported physician diagnosis (including hypertension, diabetes, dyslipidemia, and cardiovascular diseases). Body weight and height were measured twice at baseline by trained staff using a vertical weight scale and a metric scale, according to standardized protocols and the readings were recorded to the nearest 0.1 kg and 0.1 cm, respectively. Body mass index (BMI) was calculated as weight (kilogram) divided by height (meter) squared based on the measurement.

### Assessment of outcomes

MDD and GAD were measured using PHQ-2 and GAD-2 questionnaires, which were initially developed by Kroenke, Spitzer and their colleagues in 2003 and 2007, respectively [13, 14]. As ultra-short screening tools, PHQ-2 and GAD-2 met the demands of busy primary care practice and large population-based surveys. The PHQ-2 or GAD-2 are two-item screening questionnaires based on the Diagnostic and Statistical Manual of Mental Disorders-Fourth Edition (DSM-IV) criteria to assess the frequency of self-reported depressive or anxious symptoms in the past two weeks (not at all = 0, several days = 1, more than half the days = 2, and nearly every day = 3).. The two scales have shown reasonably good psychometric properties for screening MDD and GAD in different populations [13–16]. More importantly, the GAD-2 scale displayed excellent property for identifying GAD at a cutoff of 3 in the Henan Rural Cohort Study, with a sensitivity of 0.865 and a specificity of 0.980 [16].

It is worth noting in this study that the PHQ-2 and GAD-2 scales are useful screening measures rather than diagnostic tools, so the prevalence of MDD and GAD may be overestimated. However, the differences between the “diagnosed” prevalence and the “screened” rates may not be significant due to the high sensitivity and specificity of the PHQ-2 and GAD-2. In busy primary care or large population studies, these two scales were quite suitable for saving time while still providing accepted diagnostic performances. In this study, a cutoff of 3 was adopted to assess the prevalence of MDD and GAD. In other words, participants who reported a score of 3 or above for PHQ-2 or GAD-2 scales were classified as having MDD or GAD, respectively.

### Quality control

A stringent quality assurance and quality control program was conducted to ensure good validity and reliability of study data. In this study, all the research staff were well trained to administer standardized questionnaires and instruments for data collection. All laboratory equipment were calibrated and all data were double entered in the EpiData software. In addition, some of the respondents were interviewed again after several days by the research staff for face­to­face checking.

### Statistical analysis

Continuous variables presented as mean ± standard deviation (SD) were compared using the t-test or analysis of variance, while categorical variables presented as numbers and proportions were compared using the chi-square test. Data were weighted to adjust for oversampling, nonresponse and post-stratification between the survey sample and the total population. The age-standardized prevalence of MDD and GAD were calculated by subgroup and the overall population according to the Chinese Population Census 2010. Multivariate logistic regression analyses were used to identify the potential associated factors of MDD and GAD with odds ratios (*ORs*) and corresponding 95% confidence interval (*CI*) in the full model. The interaction terms for gender with other variables were also included in the logistic regression model for the overall sample. To evaluate the comorbidity between MDD and GAD, tetrachoric correlation test was used. All tests were two-tailed and a *P-*value < 0.05 was considered statistically significant. All statistical analyses were performed using SAS 9.1 software package (SAS Institute, USA).

## Results

### Demographic characteristics

Table 1 presents the demographic characteristics of the 29,993 participants aged 18–79 years according to sex. In this study, 12,233 men (mean age: 56.49, SD: 12.386) and 17,760 women (mean age: 54.74, SD: 12.293) were included. Of the sample population, 1792 (583 men and 1209 women) and 1659 (481 men and 1178 women) subjects were detected as having MDD and GAD, respectively.

**Table 1:** Demographic characteristics

Variable	Men						Women					
	MDD^a^ (*n* = 583)	Non-MDD (*n* = 11,650)	*P*	GAD^b^ (*n* = 481)	Non-GAD (*n* = 11,752)	*P*	MDD (*n* = 1209)	Non-MDD (*n* = 16,551)	*P*	GAD (*n* = 1178)	Non-GAD (*n* = 16,582)	*P*
Age (years), mean ± SD	56.13 ± 12.860	56.51 ± 12.362	0.474	55.97 ± 12.294	56.51 ± 12.390	0.351	56.34 ± 11.414	54.62 ± 12.347	< 0.001	55.87 ± 11.174	54.66 ± 12.365	< 0.001
Marital status, *n* (%)			0.001			0.114			0.085			0.148
Married/ cohabiting	503(86.28)	10,537(90.45)		424(88.15)	10,616(90.33)		1072(88.67)	14,929(90.20)		1047(88.88)	14,954(90.18)	
Widowed/single/divorced	80(13.72)	1114(9.55)		57(11.85)	1136(9.67)		137(11.33)	1622(9.80)		131(11.12)	1628(9.82)	
Education level, *n* (%)			< 0.001			0.022			< 0.001			< 0.001
Primary school or below	235(40.31)	3853(33.07)		184(38.25)	3904(33.22)		719(59.47)	8468(51.16)		702(59.59)	8485(51.17)	
Junior high school or above	348(59.69)	7797(66.93)		297(71.75)	7848(66.78)		490(40.53)	8083(48.84)		384(32.60)	5774(34.82)	
Per capita monthly income, n (%)			< 0.001			0.030			< 0.001			< 0.001
< 500	277(47.51)	4197(36.03)		203(42.21)	4271(36.34)		572(47.31)	5786(34.96)		528(44.82)	5830(35.16)	
500~	145(24.87)	3600(30.90)		131(27.23)	3614(30.75)		327(27.05)	5374(32.47)		325(27.59)	5376(32.42)	
≥ 1000	161(27.62)	3853(33.07)		147(30.56)	3867(32.91)		310(25.64)	5391(32.57)		325(27.59)	5376(032.42)	
Current smoking, *n* (%)	277(47.51)	5691(48.85)	0.529	229(47.61)	5739(48.83)	0.598	5(0.41)	47(0.28)	0.401	6(0.51)	46(0.28)	0.156
Current drinking, *n* (%)	188(32.25)	4696(40.31)	< 0.001	166(34.51)	4718(40.15)	0.013	24(1.99)	321(1.94)	0.912	26(2.21)	319(1.92)	0.496
Physical activity, *n* (%)			0.039			0.848			0.261			0.001
Low	227(38.94)	4016(34.47)		162(33.68)	4081(34.73)		379(31.35)	4940(29.85)		304(25.81)	5015(30.24)	
Moderate	165(28.30)	3271(28.08)		140(29.11)	3296(28.05)		490(40.53)	7104(42.92)		504(42.78)	7090(42.76)	
High	191(32.76)	4363(37.45)		179(37.21)	4375(37.2)		340(28.12)	4507(27.23)		370(31.41)	4477(27.00)	
Chronic diseases history, *n* (%)	369(63.29)	7294(62.61)	0.739	293(60.91)	7370(62.71)	0.424	760(62.86)	9649(58.30)	0.002	723(61.38)	9686(58.41)	0.046
BMI^c^ (kg/m2), mean ± SD	23.89 ± 3.547	24.52 ± 3.459	< 0.001	23.95 ± 3.367	24.51 ± 3.468	< 0.001	24.68 ± 3.754	24.92 ± 3.618	0.037	24.49 ± 3.612	24.93 ± 3.627	< 0.001

^a^*MDD*: major depressive disorder, ^b^*GAD* generalized anxiety disorder, ^c^*BMI* body-mass index

### Prevalence

**Fig. 1:**
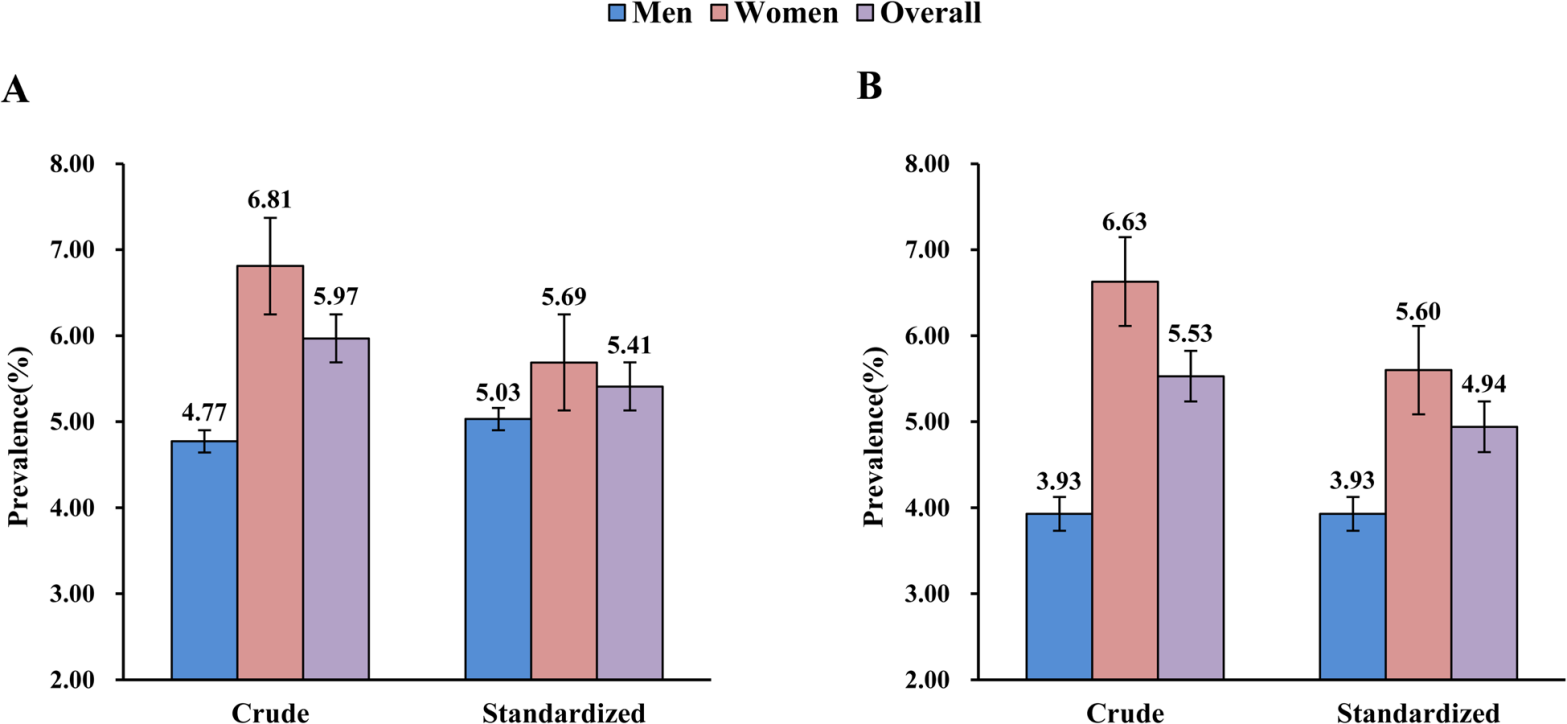
The crude and standardized prevalence of Major Depressive Disorder (**a**) and Generalized Anxiety Disorder (**b**)

Figure 1 summaries the crude and standardized prevalence of MDD and GAD, respectively. For the overall population, the crude prevalence (and 95% *CI*) of MDD and GAD were 5.97% (5.71–6.25%) vs 5.53% (5.28–5.80%) and the corresponding age-standardized prevalence were 5.41% (5.17–5.66%) vs 4.94% (4.71–5.18%), respectively. The sex-special prevalence of MDD and GAD were 4.77% (4.40–5.16%) vs 3.93% (3.59–4.29%) in men and 6.81% (6.44–7.19%) vs 6.63% (6.27–7.01%) in women, and the corresponding age-standardized rates were 5.03% (4.64–5.45%) vs 3.93% (3.59–4.28%) in men and 5.69% (5.38–6.01%) vs 5.60% (5.30–5.92%) in women, respectively.

Table 2 describes the prevalence of MDD and GAD among different characteristics according to sex. The prevalence of MDD and GAD increased with age in women (*P*_*trend*_ < 0.001 for MDD, *P*_*trend*_ = 0.001 for GAD) and decreased with BMI, educational level and per capita monthly income for both sexes. Women with chronic disease history had much higher prevalence of MDD and GAD with comparison to thosewomen without chronic disease history. Apart from the age groups of 18~ and 30~, the prevalence of MDD in women were higher than men in the remaining age groups, all the other demographic characteristics and lifestyles groups (*P* < 0.001). Similarly, the prevalence of GAD was significantly higher in women than in men in all demographic characteristics and lifestyles groups.

**Table 2:** Estimated prevalence (%) of major depressive disorder (MDD) and generalized anxiety disorder (GAD) among Chinese adults

Variable	Total		Men		Women	
	MDD (*n* = 583)	GAD (*n* = 481)	MDD (*n* = 583)	GAD (*n* = 481)	MDD (*n* = 1209)	GAD (*n* = 1178)
	Prevalence (95%*CI*)	Prevalence *(*95%*CI)*	Prevalence (95%*CI*)	Prevalence *(*95%*CI)*	Prevalence *(*95%*CI)*	Prevalence *(*95%*CI)*
Age						
18~	4.31 (3.09–5.53)	3.37 (2.29–4.46)	5.01 (2.86–7.16)	3.26 (1.51–5.01)	3.89 (2.43–5.35)	3.44 (2.05–4.82)
30~	5.22 (4.31–6.12)	4.91 (4.03–5.79)	5.68 (4.12–7.24)	4.26 (2.90–5.62)	4.99 (3.88–6.11)	5.27 (4.12–6.41)
40~	5.77 (5.15–6.39)	5.71 (5.10–6.33)	5.11 (4.16–6.07)	4.48 (3.58–5.37)	6.18 (5.37–6.99)	6.44 (5.62–7.27)
50~	6.04 (5.52–6.55)	6.01 (5.50–6.52)	4.32 (3.61–5.04)	4.01 (3.32–4.69)	7.14 (6.44–7.84)	7.27 (6.57–7.98)
60~	6.12 (5.63–6.61)	5.35 (4.89–5.81)	4.47 (3.83–5.11)	3.63 (3.05–4.21)	7.46 (6.740–8.18)	6.75 (6.06–7.44)
70~	6.50 (5.70–7.29)	5.58 (4.84–6.32)	5.34 (4.29–6.40)	3.85 (2.95–4.76)	7.68 (6.51–8.85)	7.07 (5.95–820)
*P*	0.053	0.006	0.350	0.638	< 0.001	0.001
*P*_*trend*_	0.002	0.125	0.559	0.380	< 0.001	0.001
Marital status						
Married/ cohabiting	5.82 (5.55–6.10)	5.43 (5.16–5.70)	4.56 (4.17–4.95)	3.84 (3.48–4.20)	6.70 (6.31–7.09)	6.54 (6.16–6.93)
Widowed/single/divorced	7.35 (6.41–8.29)	6.37 (5.49–7.26)	6.71 (5.28–8.13)	4.78 (3.57–5.99)	7.79 (6.53–9.04)	7.45 (6.22–8.68)
*P*	0.001	0.036	0.001	0.114	0.085	0.148
Education level						
Primary school or below	7.19 (6.75–7.63)	6.67 (6.24–7.09)	5.75 (5.03–6.46)	4.50 (3.87–5.14)	7.83 (7.28–8.38)	7.64 (7.10–8.18)
Junior high school or above	5.01 (4.68–5.34)	4.62 (4.31–4.94)	4.27 (3.83–4.71)	3.65 (3.24–4.05)	5.72 (5.22–6.21)	5.55 (5.07–6.04)
*P*	< 0.001	< 0.001	< 0.001	0.022	< 0.001	< 0.001
Per capita monthly income						
< 500	7.80 (7.29–8.30)	6.75 (6.28–7.23)	6.19 (5.48–6.90)	4.54 (3.93–5.15)	9.00 (8.29–9.70)	8.30 (7.63–8.98)
500~	4.97 (4.53–5.41)	4.80 (4.37–5.23)	3.87 (3.25–4.49)	3.50 (2.91–4.09)	5.74 (5.13–6.34)	5.70 (5.10–6.30)
≥ 1000	4.83 (4.40–5.26)	4.86 (4.43–5.29)	4.01 (3.40–4.62)	3.66 (3.08–4.24)	5.44 (4.85–6.03)	5.70 (5.10-)6.30
*P*	< 0.001	< 0.001	< 0.001	0.030	< 0.001	< 0.001
*P*_*trend*_	< 0.001	< 0.001	< 0.001	0.034	< 0.001	< 0.001
Current smoking						
No	6.26 (5.96–6.57)	5.93 (5.63–6.23)	4.88 (4.35–5.42)	4.02 (3.54–4.51)	6.80 (6.43–7.17)	6.62 (6.25–6.98)
Yes	4.68 (4.15–5.21)	3.91 (3.42–4.40)	4.64 (4.11–5.18)	3.84 (3.35–4.32)	9.62 (1.33–17.90)	11.54 (2.56–20.52)
*P*	< 0.001	< 0.001	0.529	0.598	0.421	0.155
Current drinking						
No	6.35 (6.04–6.65)	5.91 (5.62–6.21)	5.37 (4.86–5.89)	4.29 (3.82–4.75)	6.80 (6.43–7.18)	6.62 (6.25–6.98)
Yes	4.04 (3.51–4.58)	3.68 (3.17–4.19)	3.85 (3.31–4.39)	3.40 (2.89–3.91)	6.96 (4.26–9.65)	7.54 (4.74–10.34)
*P*	< 0.001	< 0.001	< 0.001	0.013	0.912	0.496
Physical activity						
Low	6.35 (5.86–6.84)	4.87 (4.44–5.31)	5.35 (4.67–6.03)	3.82 (3.24–4.39)	7.13 (6.43–7.82)	5.72 (5.09–6.34)
Moderate	5.89 (5.45–6.330	5.76 (5.32–6.20)	4.80 (4.09–5.52)	4.07 (3.41–4.74)	6.45 (5.90–7.01)	6.64 (6.08–7.20)
High	5.64 (5.17–6.11)	5.80 (5.33–6.28)	4.19 (3.61–4.78)	3.93 (3.37–4.50)	7.01 (6.30–7.73)	7.63 (6.89–8.380
*P*	0.132	0.003	0.039	0.848	0.261	0.001
*P*_*trend*_	0.045	0.004	0.011	0.793	0.787	< 0.001
Chronic diseases history						
No	5.52 (5.11–5.93)	5.31 (4.90–5.71)	4.68 (4.07–5.30)	4.11 (3.54–4.69)	6.11 (5.56–6.66)	6.19 (5.64–6.74)
Yes	6.25 (5.89–6.60)	5.61 (5.27–5.95)	4.82 (4.34–5.29)	3.82 (3.39–4.25)	7.30 (6.80–7.80)	6.95 (6.46–7.43)
*P*	0.014	0.398	0.739	0.424	0.002	0.046
Body-mass index						
Underweight	6.22 (5.80–6.65)	5.91 (4.24–7.56)	8.19 (5.27–11.11)	5.85 (3.35–8.35)	9.15 (6.44–11.87)	5.95 (3.72–8.18)
Normal	8.73 (6.75–10.71)	6.07 (5.65–6.49)	5.23 (4.63–5.82)	4.33 (3.78–4.87)	6.98 (6.39–7.58)	7.39 (6.78–8.01)
Overweight	5.60 (5.19–6.02)	5.34 (4.93–5.75)	4.37 (3.78–4.96)	3.75 (3.20–4.30)	6.42 (5.84–6.99)	6.39 (5.82–6.96)
Obesity	5.62 (4.99–6.26)	4.56 (3.99–5.14)	3.63 (2.78–4.48)	2.93 (2.17–3.70)	6.79 (5.92–7.66)	5.51 (4.72–6.30)
*P*	0.001	0.001	< 0.001	0.012	0.120	0.003
*P*_*trend*_	0.003	< 0.001	< 0.001	0.001	0.180	0.001

**Fig. 2:**
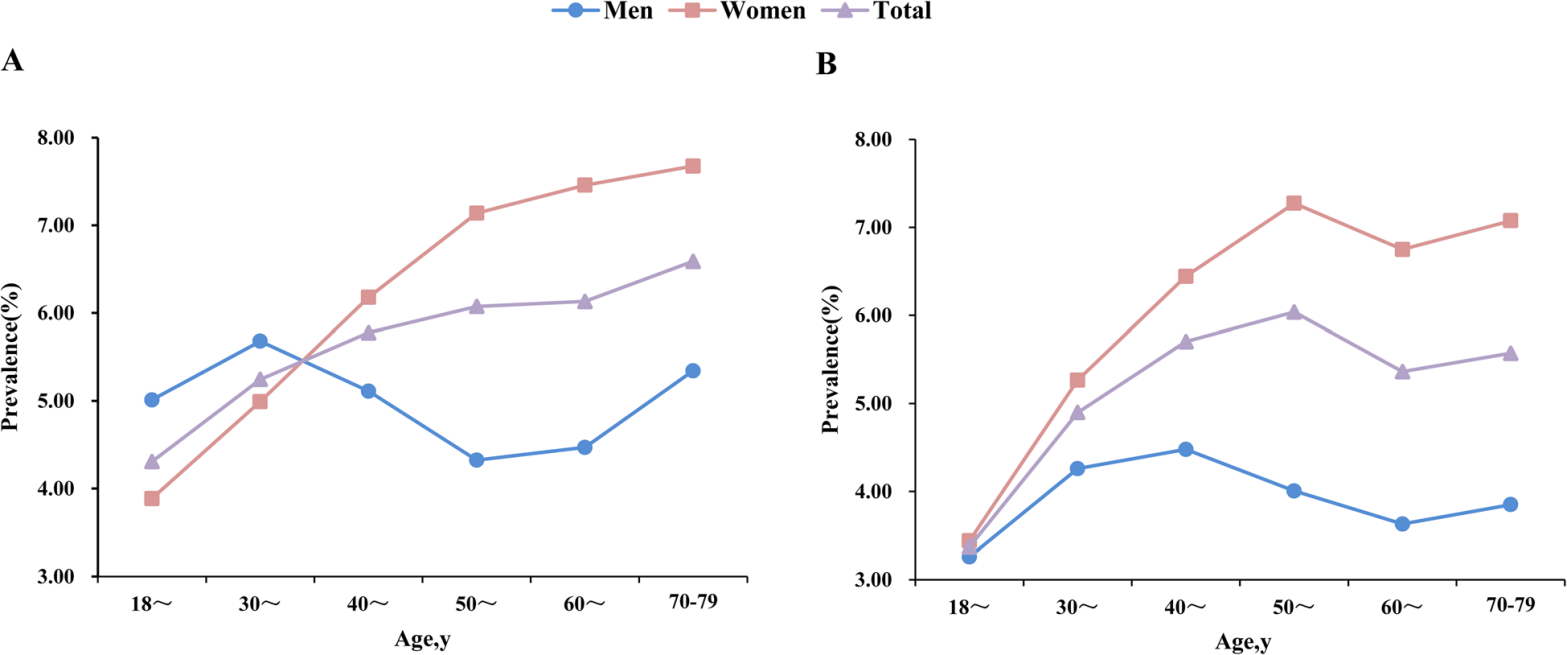
The prevalence of Major Depressive Disorder (**a**) and Generalized Anxiety Disorder (**b**) with age in different sexes

Figure 2 shows the trends of age-standardized prevalence of MDD and GAD with age in different sexes. The prevalence of MDD tended to increase with age for women while it tended to decrease for 35–55-year-old men. Men had a higher prevalence of MDD than women at age younger than 40 years, but lower at an older age (Fig. 2a). The prevalence of GAD increased with age until 45 years in men and 55 years in women, respectively and then decreased for both sexes. By the age of 65, there was a slight recovery. In general, the prevalence of GAD in women was higher than that in men across all age groups (Fig. 2b).

### Comorbidity

**Fig. 3:**
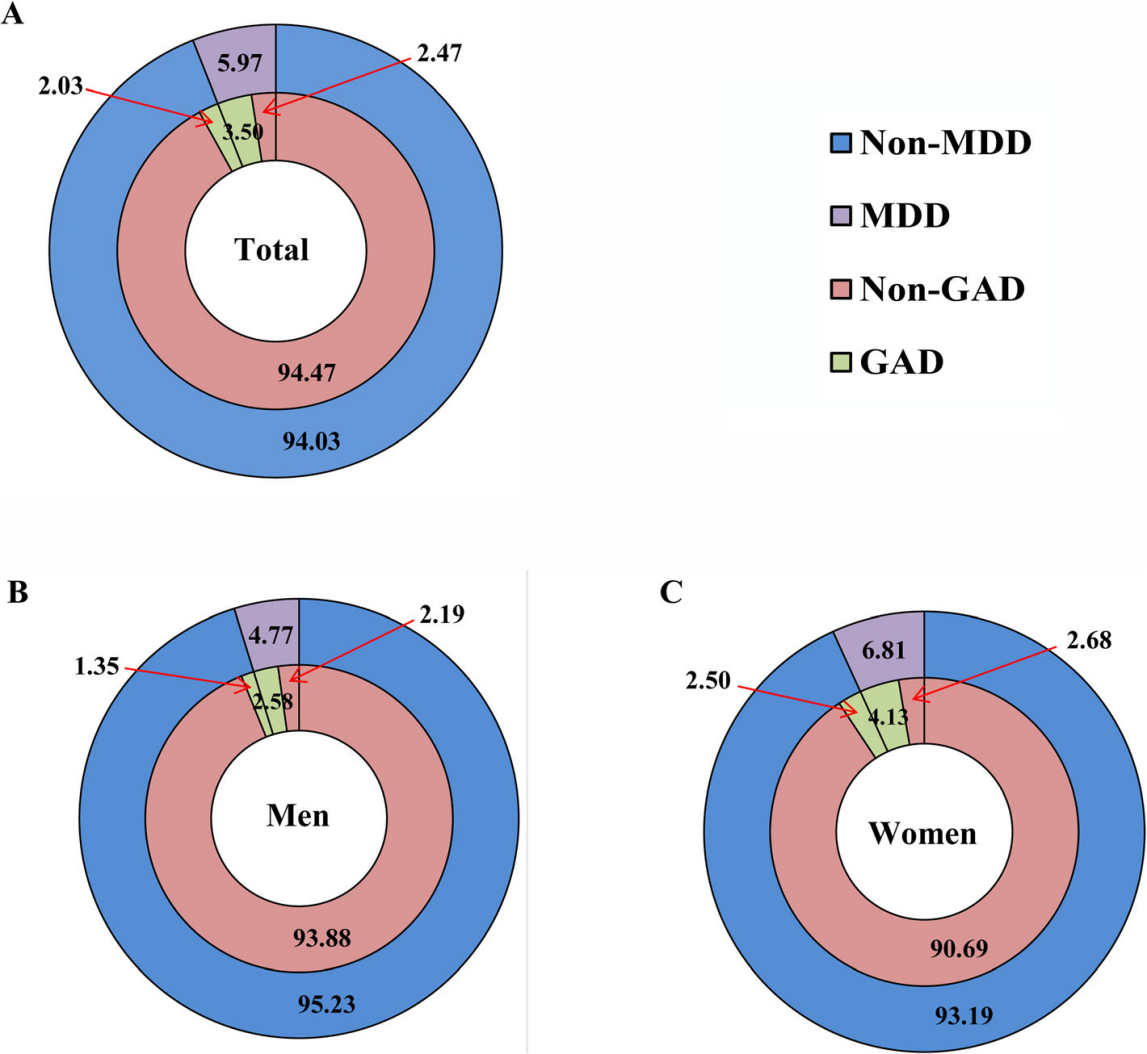
The percentage distribution of Major Depressive Disorder and Generalized Anxiety Disorder in total (**a**), men (**b**) and women (**c**)

Among the 29,993 participants, a total of 2402 subjects (748 men and 1654 women) had MDD or GAD, and the corresponding age-standardized prevalence was 8.01% (6.11% in men and 9.31% in women). In detail, 743, 610, and 1049 subjects were diagnosed with only MDD, only GAD, and combined MDD and GAD, and the corresponding prevalence were 2.47, 2.03, and 3.50%, respectively. About 58.63% of MDD cases showed evidence of comorbid GAD, while 63.29% of those with GAD met criteria for concurrent MDD (*r* = 0.88, *P* = 0.01). The more detailed information for the distribution of MDD and GAD in different sexes were shown in Fig. 3.

### Multivariable risk assessment

Table 3 describes the odds ratios (*OR*s) and corresponding 95% *CI* of potential associated factors of MDD and GAD in the full model according to sex. The multivariate logistic regression analyses showed that being women, widowed/single/divorced, non-current drinkers, having an educational level below junior high school, a low per capita monthly income, and chronic disease history were all significantly associated with increased risks of MDD or GAD in the overall population. Women had significant age-related risks of MDD and GAD while men did not. Specifically, women at 40~60 years of age were more likely to suffer from MDD or GAD compared with those aged 18~30 years. Besides, the interaction between gender and age for MDD was statistically significant with a *P*-value of 0.001. Remarkably, there were significant associations between overweight or/and obesity and decreased risks of MDD and GAD in both men and women.

**Table 3:** Association between potential risk factors and major depressive disorder (MDD) and generalized anxiety disorder (GAD)

Variable	Total		Men		Women	
	MDD (*n* = 583)	GAD (*n* = 481)	MDD (*n* = 583)	GAD (*n* = 481)	MDD (*n* = 1209)	GAD (*n* = 1178)
	*OR* (95% *CI*)	*OR* (95% *CI*)	*OR* (95% *CI*)	*OR* (95% *CI*)	*OR* (95% *CI*)	*OR* (95% *CI*)
Age						
18~	1.00	1.00	1.00	1.00	1.00	1.00
30~	1.27 (0.89–1.81)	1.48 (1.01–2.18)^*^	1.33 (0.77–2.31)	1.48 (0.76–2.85)	1.28 (0.81–2.03)	1.51 (0.93–2.43)
40~	1.31 (0.94–1.81)	1.59 (1.11–2.28)^*^	1.10 (0.66–1.84)	1.47 (0.80–2.70)	1.46 (0.95–2.24)	1.64 (1.05–2.56)^*^
50~	1.36(0.99–1.86)	1.67 (1.17–2.37)^*^	0.86 (0.52–1.41)	1.24 (0.68–2.26)	1.66 (1.09–2.53)^*^	1.81 (1.16–2.81)^*^
60~	1.15 (0.83–1.59)	1.27 (0.89–1.82)	0.70 (0.42–1.16)	0.95 (0.52–1.73)	1.45 (0.94–2.23)	1.38 (0.88–2.17)
70~	1.06 (0.75–1.48)	1.23 (0.85–1.80)	0.67 (0.40–1.14)	0.89 (0.47–1.67)	1.30 (0.83–2.04)	1.36 (0.85–2.19)
*P*_*trend*_	< 0.001	< 0.001	< 0.001	< 0.001	0.574	0.592
*P*_*interaction*_	0.001	0.161				
Sex						
Men	1.00	1.00				
Women	1.30 (1.13–1.49)^*^	1.54 (1.33–1.80)				
Marital status						
Married/ cohabiting	1.00	1.00	1.00	1.00	1.00	1.00
Widowed/single/divorced	1.22 (1.05–1.43)^*^	1.19 (1.01–1.41)^*^	1.37 (1.06–1.77)^*^	1.22 (0.91–1.65)	1.11 (0.91–1.35)	1.15 (0.94–1.41)
Education level						
Primary school or below	1.00	1.00	1.00	1.00	1.00	1.00
Junior high school or above	0.76 (0.68–0.84)^*^	0.72 (0.62–0.84)^*^	0.75 (0.62–0.90)^*^	0.79 (0.64–0.97)^*^	0.79 (0.69–0.91)^*^	0.73 (0.64–0.84)^*^
Per capita monthly income						
< 500	1.00	1.00	1.00	1.00	1.00	1.00
500~	0.63 (0.56–0.71)^*^	0.73 (0.65–0.83)^*^	0.62 (0.51–0.77)^*^	0.77 (0.61–0.97)^*^	0.64 (0.55–0.74)^*^	0.69 (0.59–0.80)^*^
≥ 1000	0.63 (0.56–0.72)^*^	0.78 (0.68–0.88)^*^	0.66 (0.54–0.81)^*^	0.82 (0.65–1.03)	0.62 (0.54–0.72)^*^	0.70 (0.61–0.82)^*^
*P*_*trend*_	< 0.001	0.069	< 0.001	0.069	< 0.001	< 0.001
Current smoking						
No	1.00	1.00	1.00	1.00	1.00	1.00
Yes	0.99 (0.84–1.18)	0.95 (0.78–1.14)	0.97 (0.82–1.16)	0.95 (0.78–1.14)	1.26 (0.50–3.20)	1.59 (0.67–3.77)
Current drinking						
No	1.00	1.00	1.00	1.00	1.00	1.00
Yes	0.81 (0.68–0.96)^*^	0.88 (0.74–1.06)	0.75 (0.62–0.91)^*^	0.80 (0.66–0.98)^*^	1.07 (0.70–1.63)	1.13 (0.75–1.71)
Physical activity						
Low	1.00	1.00	1.00	1.00	1.00	1.00
Moderate	0.88 (0.78–0.99)^*^	1.11 (0.97–1.26)	0.88 (0.72–1.09)	1.04 (0.82–1.31)	0.91 (0.79–1.05)	1.17 (1.01–1.36)^*^
High	0.89 (0.78–1.01)	1.20 (1.05–1.37)^*^	0.79 (0.65–0.97)^*^	1.01 (0.81–1.26)	0.95 (0.81–1.11)	1.31 (1.11–1.54)^*^
*P*_*trend*_	0.070	0.003	0.022	0.942	0.479	0.001
Chronic diseases history						
No	1.00	1.00	1.00	1.00	1.00	1.00
Yes	1.11 (1.01–1.24)^*^	1.09 (0.97–1.22)	1.13 (0.94–1.36)	1.03 (0.84–1.25)	1.12 (0.98–1.28)	1.12 (0.98–1.28)
Body-mass index						
Underweight	1.43 (1.09–1.87)^*^	1.01 (0.73–1.37)	1.49 (0.99–2.25)	1.34 (0.83–2.15)	1.41 (1.01–1.98)^*^	0.85 (0.57–1.29)
Normal	1.00	1.00	1.00	1.00	1.00	1.00
Overweight	0.87 (0.78–0.97)^*^	0.84 (0.75–0.94)^*^	0.84 (0.69–1.01)	0.86 (0.70–1.06)	0.87 (0.76–0.99)^*^	0.81 (0.71–0.93)^*^
Obesity	0.86 (0.74–0.99)^*^	0.70 (0.60–0.82)^*^	0.66 (0.50–0.88)^*^	0.66 (0.48–0.90)^*^	0.90 (0.76–1.07)	0.69 (0.57–0.82)^*^
*P*_*trend*_	0.001	0.003	< 0.001	0.003	0.023	0.001

^*^P < 0.05; *P*_interaction_: the interaction term between gender and age

## Discussion

This study revealed comparatively low prevalence rates of MDD and GAD in a Chinese rural population. According to the data from the National Health and Nutrition Examination Survey, 8.1% of American adults had major depression over 2 weeks during 2013–2016 [17], which was much higher than the prevalence in this study (5.41%). Wittchen et al. reported that the prevalence of major depression and anxiety disorders were 6.9 and 14.0% in Europe 2010, respectively [18]. To some extent, it accorded with the previous finding that the prevalence of GAD was lower in Asian samples than in Western samples [19]. However, the prevalence of MDD and GAD found in the current study were on the higher bound of previously reported estimates for the general population of other Asian countries [20–22]. It indicated the importance of MDD and GAD as serious public health problems in China. There were also a large number of other reports on the prevalence of MDD and GAD in China. The data from the China National Health and Wellness Survey showed that the prevalence of MDD and GAD in Chinese urban population were 6.0 and 5.3%, respectively [23, 24]. It seemed that the prevalence in rural areas presented in this study were a little lower compared with those in urban areas although it may not be completely comparable due to the different screening methods. This phenomenon might be attributed to the high pressure from tight job schedules, high housing costs, and medical difficulties in urban areas, especially in the big cities [25]. Nevertheless, given the large population in rural areas, people in rural China are suffering from a serious burden of MDD and GAD.

Comorbid MDD and GAD was additionally assessed for its great importance in psychiatric practice and epidemiology. In the current study, MDD and GAD had a strong correlation (*r* = 0.88, *P* = 0.01). The high comorbidity has been investigated in different populations [26, 27] and the twin study provided evidence in the area of genetics [28]. In addition, the nosologic relationship and overlap between MDD and GAD contributed to the high comorbidity to some extent [29]. Consequently, the high comorbidity between MDD and GAD was considered to do more harm to human health than either MDD or GAD did. Cairney’s research indicated that significantly lower well-being and greater impairment were found in adults with two or more disorders compared with those who had no or one mental disorder [30]. Therefore, early successful management of adults with MDD or/and GAD might prove critical to averting such a development.

Higher prevalence of MDD and GAD among women have been observed previously [10, 26], and similar results were discovered in this study. Consistent with previous epidemiological researches [8–10], women were 1.30 and 1.54 times more likely to suffer from MDD and GAD than men after controlling for potential confounding factors, respectively. The current study also provided information on the other associated factors besides sex. In accord with previous studies [31–35], our results found that being unmarried, having low educational level and low per capita monthly income were significantly associated with increased risks of MDD and GAD. In addition, an increased risk of MDD was found in women of 50~60 years old and an increased risk of GAD was found in women of 40~60 years old whereas the age-related risks were not discovered in men. The presence of the significant interaction (*P* = 0.001) also indicated that the effects of age on MDD was different in disparate genders. The sex discrepancies in age-related risks of MDD and GAD have been clearly proven in previous studies, which showed that menopause transition was a period of heightened risk for depression and anxiety among women [36–38]. However, the associations between chronic disease history and MDD and/or GAD became borderline nonsignificant after adjustment for socio-demographic factors in our study, whereas the associations existed in the unadjusted model (Table 2). In general, chronic diseases were consistently discovered to be robustly associated with MDD and/or GAD in most epidemiological studies [32, 35, 39]. The inconsistent results may be attributed to the different defined criteria of chronic diseases, in which information in this study was only based on self-report rather than a more accurate hospital or insurance system data. Future work should be done to verify the cases of chronic diseases.

Contrary to the results of previous studies, the current result showed that moderate and high levels of physical activity were associated with slightly increased risks of GAD in the overall sample and in the women population. This may be due to the vulnerability to mental illness when pursuing vigorous exercise [40], especially among women whose physical fitness are not so good as that of men. Besides, the GAD patients often tended to be too restless to sit still thus would contribute to the physical activity. Finally, the cross-sectional nature of this study and potential reverse causation bias might partly explain the inverse associations. We cannot judge the time order between physical activity and GAD. Further longitudinal studies are needed to explicate the mechanisms between physical activity and GAD.

The relationships between overweight/obesity and depression/anxiety are not consistent in most previous studies. Strongly consistent with the so-called ‘jolly fat’ hypothesis [41], our results showed that BMI was significantly inversely associated with MDD and GAD. The Korea National Health and Nutrition Examination Survey and China Health and Retirement Longitudinal Study also reported that overweight/obesity significantly lowered the risk of depression [42, 43]. This inverse association may be related to Chinese traditional culture in which Chinese people have positive perceptions towards obesity that being moderately fat is a symbol of health. However, the logistic regression model did not take full account of the interactions among the associated factors. For example, people with overweight/obesity were more likely to have high education, high income and be married (all *P* < 0.001) which benefited mental health. In general, the true relationships between overweight/obesity and depression/anxiety in Chinese need further exploration.

The above analysis on risk factors associated with MDD and GAD could provide important evidence in the preventive strategies. The society and government should pay more attention to women, widowed/single/divorced individuals, and those with low education, low income, or chronic diseases. Taking moderate exercise and improving physical health may be beneficial in preventing and controlling depression and anxiety in all participants. In a word, it is essential to closely monitor and control high-risk factors of MDD and GAD in the rural areas of China.

The present study has several strengths. Firstly, this study was conducted in a large sample of the general population in rural China. Secondly, a range of potential confounding variables were included in the analysis. Therefore, we were able to assess the associations between these factors and the prevalence of depression and anxiety. In addition, the validity and reliability were guaranteed through the strict quality control mechanism at every stage of the research.

There are also several limitations in this study. Firstly, the PHQ-2 and GAD-2 are useful screening measures rather than diagnostic tools [15, 16], thus the prevalence of MDD and GAD may be overestimated. However, the differences between the diagnosed prevalence and the screened rates were not statistically significant according to Bolton et al. [44] and these two screening tools may largely reflect the psychological status of the subjects. Secondly, the “drinking status” might not be appropriately categorized and the types, frequency, amounts, and occasions of drinking should be taken into consideration. Maybe this was the reason why the current drinkers were less depressive and anxious in the present study. Further study is deemed necessary concerning more detailed analyses of the drinking. Thirdly, the lack of information on employment status in the logistics regression models may affect our results according to previous studies. Finally, due to the cross-sectional nature of this study and potential reverse causation bias, associations between some risk factors and MDD or GAD were in unexpected directions.

## Conclusion

The finding showed comparatively lower prevalence rates of MDD and GAD in a Chinese rural population than urban people. The gender differences in both MDD and GAD indicated that women deserved more attention. Besides, effective interventions on the modifiable associated factors are urgently needed to improve the mental conditions for Chinese rural population.

## Data Availability

The datasets used and/or analyzed during the current study are available from the corresponding author on reasonable request.

## Abbreviations

BMI: Body mass index
CI: Confidence interval
DSM-IV: Diagnostic and statistical manual of mental disorders-fourth edition
GAD: Generalized anxiety disorder
GAD-2: Generalized anxiety disorder-2
IPAQ: International physical activity questionnaire
MDD: Major depressive disorder
ORs: Odds ratios
PHQ-2: Patient health questionnaire-2
SD: Standard deviation
